# Why lockdown of the elderly is not ageist and why levelling down equality is wrong

**DOI:** 10.1136/medethics-2020-106336

**Published:** 2020-06-19

**Authors:** Julian Savulescu, James Cameron

**Affiliations:** 1 Faculty of Philosophy, Uehiro Centre for Practical Ethics, University of Oxford, Oxford, UK; 2 Biomedical Ethics Research Group, Murdoch Childrens Research Institute, Parkville, Victoria, Australia; 3 Melbourne Law School, The University of Melbourne, Carlton, Victoria, Australia

**Keywords:** aged, ethics, philosophical ethics, public health ethics, public policy

## Abstract

In order to prevent the rapid spread of COVID-19, governments have placed significant restrictions on liberty, including preventing all non-essential travel. These restrictions were justified on the basis the health system may be overwhelmed by COVID-19 cases and in order to prevent deaths. Governments are now considering how they may de-escalate these restrictions. This article argues that an appropriate approach may be to lift the general lockdown but implement selective isolation of the elderly. While this discriminates against the elderly, there is a morally relevant difference—the elderly are far more likely to require hospitalisation and die than the rest of the population. If the aim is to ensure the health system is not overwhelmed and to reduce the death rate, preventing the elderly from contracting the virus may be an effective means of achieving this. The alternative is to continue to keep everyone in lockdown. It is argued that this is levelling down equality and is unethical. It suggests that in order for the elderly to avoid contracting the virus, the whole population should have their liberty deprived, even though the same result could be achieved by only restricting the liberty of the elderly. Similar arguments may also be applied to all groups at increased risk of COVID-19, such as men and those with comorbidities, the obese and people from ethnic minorities or socially deprived groups. This utilitarian concern must be balanced against other considerations, such as equality and justice, and the benefits gained from discriminating in these ways must be proportionately greater than the negative consequences of doing so. Such selective discrimination will be most justified when the liberty restriction to a group promotes the well-being of that group (apart from its wider social benefits).

## Is selective lockdown of the elderly (and other high-risk groups) justified discrimination?

Countries all around the world are struggling to develop policies on how to exit lockdown for COVID-19 to restore liberty and prevent economic collapse, while also protecting public health from a resurgence of the pandemic. Hopefully, an effective vaccine or treatment will emerge but in the meantime, the strategy involves continued containment and management of limited resources.

One strategy is a staged relaxation of lockdown. This article explores whether a selective continuation of lockdown on certain groups, in this case the aged, represents unjust discrimination. It is argued that selective isolation of the elderly is a more proportionate response to the risks posed than continuing a complete lockdown. The purpose of the lockdown is to limit deaths resulting from COVID-19 and to prevent the healthcare system from being overwhelmed. The elderly face a significantly higher risk of becoming severely unwell and dying if they contract COVID-19.^1^ This not only means the elderly gain more by not contracting the virus, but that they pose the greatest risk to society because the elderly are more likely to place significant strain on limited intensive care resources. To continue to require everyone to be locked down is to engage in levelling down equality. This is because for the elderly to experience the benefit of not contracting COVID-19 everyone must experience the same deprivation of liberty, even though everyone else will not experience the same benefit and does not pose the same risk of overwhelming the healthcare system.

If such arguments are accepted in relation to the elderly, it must also be recognised that there are other groups who may also be identified as facing a higher risk of becoming severely unwell and dying from COVID-19. This includes men, the obese, those with comorbidities, some ethnic minorities and people with particular disabilities.^2^ But it is not sufficient to simply recognise a relevant difference and that a benefit may be derived from discriminating. The benefit gained must be proportionately greater than the negative consequences of discriminating, which undermines equality and in some circumstances may be particularly unjust.

## The ideal of equality and the concept of unjust discrimination

Aristotle described the principle of equality as treating like cases alike, unless there is a morally relevant difference.^3^ For example, if men are allowed to vote, and women are not, the only difference is sex. Sex cannot of itself make a difference to the capacity or performance of voting—it involves a mere chromosomal or anatomical difference. Unless one could point to an inherent property that tracked with sex that affected ability to vote, then this violates Aristotle’s principle and is unjust discrimination.

Discrimination is not always unjust. If there is a morally relevant difference, it may be acceptable. The government invests millions in screening women for breast cancer, but not men, even though breast cancer does occur in men. The reason for this is that breast cancer is much more likely in women. So, you will save more lives with the limited resource the government has available for prevention and treatment of breast cancer if you (justly) discriminate between men and women in this way.

This is not sexist because there is a morally relevant difference sufficient to justify different treatment: the probability of developing breast cancer. However, if there were a better, more accurate proxy for breast cancer risk besides sex, say some genetic mutation, then to continue to discriminate on the basis of sex would be unjust, all else being equal. There are morally relevant differences that justify different treatment, as Plato recognised, ‘indiscriminate equality for all amounts to inequality’.^4^

## Isolation and quarantine

The same principles apply to isolation and quarantine. Currently, those who are quarantined are those most likely to have been in contact with a pathogen. Early on in the COVID-19 pandemic, those who had been in contact with someone with COVID-19 or who had travelled were tested, then later isolated. This is because they were statistically more likely to have COVID-19. This was not unjust discrimination against those who had travelled because there was a morally relevant difference, there was a greater likelihood of them having the virus, which meant a greater likelihood of infecting others.

## Selective isolation of the elderly

Currently lockdown aims to ‘flatten the curve’. Some countries, like New Zealand, have eradicated it but this seems very unlikely in countries like the UK where there is already a significant pool of infected individuals and prolonged border closure is unlikely to be effective or tolerated. The aim is to slow infection so that hospitals are not overwhelmed until a vaccine or treatment arrives. However, this can also be achieved by preventing those most likely to become ill from becoming ill: those who are elderly or who have relevant comorbidities (we will not mention those with comorbidities further but the same principles apply to them).

One possible strategy for de-escalation of the lockdown is to allow those in the workforce to return to work and for social life to return, but to continue to isolate the elderly. Call this selective isolation of the elderly. This would not be permanent but temporary until there is sufficient herd immunity or a vaccine/treatment emerges.

We will not attempt to identify an appropriate cut-off to identify ‘the elderly’ here. It will have to correlate with the elbow of an exponential curve and be easily enforceable. It is clear though that COVID-19 poses a significantly increased risk of hospitalisation and death to the elderly when compared with the rest of the population. One study of cases in China found that while 1% of people in their 20s who contracted the virus would require hospitalisation, 18.4% of those over 80 would. Similarly, while the estimated overall case fatality ratio was 1.38%, this was 13.4% in people aged over 80.^1^ Current data for England and Wales show that 89% of deaths are in those over 65 and 47% of these deaths are in those over 85.^5^

Sweden has imposed relatively few restrictions but has asked citizens to avoid non-essential travel and asked those over 70 to stay at home.^6^ The approach has been controversial and the death rate in Sweden has been higher than some neighbouring countries, though this has been mainly due to outbreaks in aged care facilities.^7^ But the healthcare system has also not been overburdened. Also, Sweden has not implemented strict isolation rules for the elderly, and only limited specific protections to ensure they may remain isolated. A number of issues have also been identified with the practical management of age care facilities in Sweden.^8^

If those most likely to get seriously ill and require hospitalisation do not get ill (or are not hospitalised), it may be possible to avoid overburdening the health system until herd immunity is achieved or a vaccine arrives. Rather than simply slowing the infection, selective isolation of the elderly would slow the infection among those most likely to overburden the health system.

## Selective isolation of the elderly and ageism

Some have claimed that selective isolation of the elderly would constitute unjust discrimination against the elderly. In a letter published in the *Daily Mail*, former Home Secretary David Blunkett suggests ‘[a]ll it would do is divide society on grounds of age—and that is as wrong as separating people because of their race or gender….I don’t know of any evidence to suggest that pensioners spread COVID-19 more virulently than younger people either’.^9^

But the issue, unlike usual quarantine, is not their spreading the virus but the probability that they will require National Health Service (NHS) resources if they do become ill. It is not only a question of direct harm to others, but indirect harm to others through the use of a limited resource.

The recent backlash against suggestions that if there is a shortage of intensive care unit beds elderly patients with COVID-19 should be deprioritised to maximise the benefit derived from limited resources demonstrates the concern that exists with discrimination on the basis of age. For example, Harris argues that it is impermissible to differentiate between patients based on survival rates and life expectancy as the value of each individual’s remaining life must be valued equally.^10^ This argument suggests that age should not be considered a relevant difference, even if there would be benefits in discriminating on this basis, because doing so would undermine the principle that all human lives should be respected equally. This argument suggests that in some instances, it is not acceptable to weigh equality against achieving other benefits. These arguments are made in relation to valuing lives equally, but could also be applied to valuing liberty equally, that it is not acceptable to privilege some people’s liberty over others’.

So is it unjust discrimination to selectively isolate those most likely to get sufficiently ill to need a limited public resource? No, it is analogous to only screening women for breast cancer on the basis of their higher probability of getting sick.

It is using age like sex, as a basis for a medical decision because that feature correlates with a robust statistically higher likelihood of getting seriously ill. That feature is the best available proxy, given the efficiency limitations on systematically screening for a more nuanced risk factor for a morally relevant outcome—the likelihood of getting seriously ill. Isolating only the elderly for COVID-19 is no more ageist than only screening women for breast cancer is sexist.

One might object that breast cancer screening is voluntary while lockdown is not. Indeed, the real issue is coercion and loss of liberty. Selective isolation appears to punish those who are vulnerable to the disease by placing them at a relative disadvantage. This is discussed further below. For the present, it is worth noting that if coercion is bad, it is worse if more people are coerced (complete lockdown) than if fewer are (selective isolation of the elderly). Further, coercion is used in standard quarantine on the basis of risk of harm to others. That is precisely the same justification that could be provided for selective isolation of the elderly, except it is indirect harm to others through consumption of limited resources.

## Proportionality

Summarising and building on Aristotle, Finnis argued that justice requires more than *simply* treating like cases alike or promoting proportional equality; it requires pursuit of a common good with recognition of ‘the interests and welfare that include many elements besides their being shared equally’.^11^ Simply identifying discrimination is not a sufficient basis to reject selective isolation, there are other relevant factors that must be weighed against its inequitable impact. Equality must still be a relevant consideration, but a measure may be justified if it would provide proportionately greater benefits and it is necessary to discriminate to achieve those benefits.

It may be argued that selective isolation of the elderly is justified discrimination because it is a proportionate means of achieving a legitimate aim. Like the current complete lockdown, the legitimate aim of selective isolation is limiting the number of deaths caused by COVID-19 and the social disruption that this will cause.

There are three justifications for why this restriction of liberty is proportionate. The first is that the benefits to others are so significant as to outweigh the loss of liberty. Any attempt to limit the effects of the virus must be weighed against the other implications these will have for society. The current complete lockdown is already having significant economic implications and these will only get worse the longer the lockdown is in place. These economic implications must not be dismissed; they also have serious health consequences. For example, there were an estimated 260 000 excess cancer deaths after the 2008 financial crisis in Organisation for Economic Co-operation and Development (OECD) countries.^12^ Selective isolation of the elderly is likely to prevent the elderly from contracting COVID-19 and so reduce morbidity and mortality without having the same economic impact as the current complete lockdown.

The second justification is that the restriction of liberty will benefit the elderly themselves: they have the greatest chance of dying and stand to gain most from the loss of liberty.

The third justification is that loss of liberty, at this point of time, is inevitable. The alternative is a loss of liberty for the old and young. Given that there needs to be some restriction of liberty (or so we are assuming), it is better that this be less rather than more (even if more loss of liberty is more equal). Selective isolation of the elderly would allow a return to a degree of normality for the majority of society. This would have far less impact on the economy and other aspects of society than a complete lockdown. Of course this would also impose a significant burden on the elderly that was not imposed on the rest of society. This burden would be proportionate when compared with the alternatives though, which is inflicting a similar burden on everyone, including the elderly. Given the options, the elderly will be in the same position regardless of the approach taken.

## Symbolic value of equality

One objection to this proposed policy is that, as Blunkett said, this risks stigmatising a group. Equality has a symbolic value. As Hellman argues, discrimination on the basis of a particular attribute is wrong because it demeans the person affected.^13^ It demonstrates a lack of respect for them as an individual. The symbolic value of equality may also be seen in the objections to discriminating on the basis of age for admission to intensive care units; there is symbolic value in valuing all lives equally and those who oppose such measures argue that this symbolic value is greater than any relative benefits. But how much should we pay for this symbolic value and to protect one group at the expense of another group?

Often quite a significant amount. I remember a few years ago my 82-year-old mother being directed into a body scanner at Heathrow. Although some staff deny it, these scanners use ionising radiation. This increases cancer risk. They reassure you by telling you it is the same amount or less than you would receive from cosmic radiation on the same flight. But flights are dangerous too. Over the whole population (billions) being screened, some small number of people probably get cancer from this exposure.

And of course, it would be easy to tell that there was virtually zero chance of my 82-year-old mother being a terrorist. You could plug in her data from several sources and come up with a probability that is next to zero (age, sex, religion, travel history, places where she has lived, etc). But we do not profile people—we expose everyone to radiation. The reason for that is equality and avoidance of stigmatisation.

David Blunkett also refers to terrorism in his letter:

‘In 2001, I was the home secretary. There was a strong feeling among my government colleagues that the country must face down terrorism the way that it faced down the Nazis during the Blitz—by being resilient…. It looked at the balance required between protecting human rights, on the one hand, and avoiding unnecessary risk and panic on the other.’^9^

This position captures beautifully the wishful thinking of ordinary ethical thinking. There is no Hollywood happy ending where everyone is a winner. Everything has upsides and downsides. Equality may have upsides but it also means increased risk of cancer, increased risks of terrorism and increased loss of life and suffering in a pandemic. You cannot have your cake and eat it too.

## Isolating the elderly and levelling down equality

Isolating the elderly is different from screening people who are statistically more likely to be terrorists at the airport. The young man who attends a radical Islamic mosque, has nothing to gain from being selected for enhanced screening. The elderly do—they are the ones most likely to die.

And there is another reason why selective isolation of the elderly is different from profiling terrorists. While the costs of screening everyone at the airport are relatively low (a very small risk of cancer), the costs of applying the lockdown to everyone, and not just the elderly, are massive. Not only directly in terms of immediate loss of well-being and jobs, delayed or forgone medical care, but also long term through possible economic collapse and subsequent effects on health and well-being.

Selective isolation of the elderly would benefit the NHS, allow economic recovery and participation in social life for the rest of the community, but it also benefits the elderly by lowering their chances of dying from COVID-19. The last is the strongest ground for a claim of proportionality.

While one reason for not profiling people for risky screening procedures is to prevent stigmatisation, it is also an example of ‘levelling down equality’. In order for there to be equality, people who could be better off are made worse off in order to achieve equality. As Parfit has famously put it, one way to achieve equality for the blind is to make everyone blind.^14^ That is what levelling down equality requires. If we cannot cure everyone’s cancer, we cure nobody’s because that will achieve equality.

## Adverse effects on the elderly

Blunkett points out that isolation can have adverse mental and physical effects on the elderly. That is surely true. He was writing before the lockdown. But now everyone is locked down experiencing those side effects. To argue that low-risk people should not be released from lockdown because of these effects on the elderly is to advocate levelling down equality. If not everyone can have the benefit, no one shall, this principle advocates.

The effects on the elderly may be worse. They may have fewer social networks and already be more isolated. And they may have only 1 or 2 years to live, so a year in isolation is a relatively greater loss. These are important considerations.

One solution is to give greater weight to liberty and tolerate its costs. One could allow the elderly to choose not to isolate while also restricting access to health resources, such as isolation or intensive care. Using age as a determinant of access to resources may be unlawful discrimination.^15^ Societies have been reluctant to embrace this strategy to avoid depleting limited health resources. They have preferred total lockdown with likely economic disaster.

## The real issue: liberty

The real issue is not equality, but liberty. Are the restrictions of liberty reasonable and proportionate? At present, everyone’s liberty is restricted. We should prefer less liberty restriction to more. Is the liberty restriction of the elderly for up to a year a reasonable restriction? That is the essential question, not one of equality. Given the benefits to them, it may well be.

The alternative must also be considered, which is that everyone’s liberty is restricted for up to a year. This is far less reasonable given that generally younger people gain much less by not contracting the virus. If they get the virus, they will generally not be as sick and face much less risk of death. Liberty restrictions on the elderly are more justified than liberty restrictions on society more generally.

## Practical challenges

It may be argued that it is impractical to isolate the elderly. That even if it was ethically appropriate to implement selective isolation of the elderly, it would not be effective in practice and so should not be implemented. This is because many of the elderly are dependent on younger generations for care and support. The challenges of isolating the elderly in practice have become apparent in Sweden, where nursing home staff, who continue to have contact with the outside world, may have introduced the virus into nursing homes.^16^ This may be particularly problematic in relation to COVID-19, as there is an extended period of incubation in which people may not show symptoms but may still be infectious.

Of course, these practical challenges do not necessarily mean it is impractical to isolate the elderly. Those working with the elderly could also be isolated, or more stringent hygiene practices could be enforced. It has been possible to implement other quarantine practices effectively during this period and it is not clear why similar practices could not be effective to isolate the elderly. It is also a question of reducing risk, not eliminating risk.

## Legal challenges

If the government did choose to enact some form of selective isolation of the elderly, it would not be limited by the restrictions on direct discrimination in the *Equality Act 2010* if it did this through an Act of Parliament. Selective liberty restrictions on portions of the population may face a range of other legal challenges that would need to be addressed though. The legality of such a measure is not resolved in this article.

## Other approaches

There are a range of other potential approaches to managing COVID-19. None of these approaches appear to be any more practical than selective isolation of the elderly. Continuing a complete lockdown might be necessary if it had a hope of eradicating the disease. Or if a vaccine were imminent. But neither of these appear likely in the near future and the economic cost of the lockdown continues to grow. The public may also become less willing to comply as time passes and the perceived individual risk shrinks.

We could allow reintegration on the basis of an antibody passport.^17^ The same arguments would apply, this is justifiable discrimination. But this would also create a perverse incentive for people in lower risk groups to contract the virus, recognising the short-term pain of being unwell may be preferable to continuing the lockdown. This may result in another significant increase in the rate at which the virus is spreading.

Another option is release from lockdown with vigorous contact tracing through apps. But this is unlikely to work in the current voluntary format: it would not work on those most likely to violate isolation (they would be least likely to choose to use an app) and would excessively restrict liberty of those at low risk (there would be massive numbers of false positives unless there were much more widespread testing).

We could allow people to ‘take responsibility’ for internalising the costs of their behaviour. We could allow them to take risks, even significant risks, but forgo certain rights to healthcare. This would preserve the limited resource (but be associated with more deaths). However, such agreements or even contracts would never be enforced in all likelihood.

In mitigating the impact of a pandemic, there are no good options. The complete lockdown approach appears to be effectively limiting the spread of COVID-19, but this cannot continue indefinitely. Selective isolation of the elderly would place a greater burden on the elderly, but they would also have the most to gain from this. The other options available would also have a number of negative consequences or appear to be impractical.

## Broader implications

If selective isolation of the elderly is accepted on the basis the elderly face a significantly higher likelihood of requiring hospitalisation and of dying if they contract COVID-19, it may be argued that a number of other categories of people should also be selectively isolated. People with certain disabilities, some ethnic minorities, the obese and even men also face a relatively higher likelihood of hospitalisation and death if they contract COVID-19.^2^ While selective isolation of the elderly may be accepted, restricting movement on the basis of sex or ethnicity appears to be problematic.

There are two related bases for differentiating between selective isolation of the elderly and similar selective isolation on the basis of ethnicity. First, the reason that there is a correlation between age and severity of a person’s COVID-19 response appears to be the link between the inevitable association between age and deterioration of physical health. The correlation between ethnicity and severity is more speculative and ethnicity only appears to be a proxy for other relevant risk factors. Second, the relevant risk factors for which ethnicity may serve as a proxy, such as obesity and poor respiratory health, are more prevalent in particular ethnic groups because of social disadvantage.^2^ To isolate people on this basis appears to place them at a further disadvantage on the basis that they are at a greater risk from the disease because of their current social disadvantage. Such an approach may reduce overall morbidity and mortality, but it would place an unjust burden on socially disadvantaged ethnic groups.

In the case of elevated risk in men, the elevation of risk is not so great as being elderly (55% of those who have died in England and Wales are men).^5^ And the social effects of locking down all men would be proportionally greater.

As this discussion illustrates, utilitarianism needs to consider all effects and be weighed against other values. It is not always appropriate to simply proceed with the course of action that will save the most lives in the short term. In some circumstances, it may be appropriate to accept higher rates of morbidity and mortality for reasons of justice or long-term well-being. Similarly, others may accept higher rates of morbidity and mortality in order for the elderly to enjoy the same liberty as others and to promote equality. This weighing exercise should be conducted explicitly though. It must be recognised that an approach that favours equality may come at the cost of greater morbidity and mortality. In this weighing exercise, the relative good achieved by each approach should be compared. In the case of selective isolation of the elderly, considering that out of those who die, an individual is 8 times more likely to be over the age of 65, there may be significant gains in discriminating.^5^ This is in comparison to a French study that found in those aged 20-29, there were 8 fatalities for every 100,000 infections^18^, not much higher than the number of people in France who die in a traffic accident (5.5) per 100,000 population^19^. The differences in severity of COVID-19 between ethnic groups and men are much smaller, meaning the gains from selective isolation would also be much smaller, while the injustice of such a policy appears to be much greater than selective isolation of the elderly.

## Conclusion

Ethically, selective isolation is permissible. It is not unjust discrimination. It is analogous to only screening women for breast cancer: selecting those at a higher probability of suffering from a disease.

Even if it were unjust discrimination, it would be proportionate because it brings benefits to the elderly and is proportionate and necessary given the grave risks to the economy and subsequent well-being of the population of an indiscriminate lockdown. To oppose selective isolation of the elderly is to engage in levelling down equality which is itself morally repugnant.

While we have focused on the elderly because they are the largest group with the most elevated risk, similar arguments could be made in relation to other groups at elevated risk such as ethnic minorities and people with particular disabilities. Such arguments illustrate the risk of focusing solely on utilitarian concerns. Instead, any potential benefits must be weighed against other negative consequences, such as injustice and inequitable treatment.
